# Humans are not fooled by size illusions in attractiveness judgements^☆^

**DOI:** 10.1016/j.evolhumbehav.2013.11.007

**Published:** 2014-03

**Authors:** Melissa Bateson, Martin J. Tovée, Hannah R. George, Anton Gouws, Piers L. Cornelissen

**Affiliations:** a Centre for Behaviour and Evolution and Institute of Neuroscience, Newcastle University, Henry Wellcome Building for Neuroecology, Framlington Place, Newcastle upon Tyne, NE2 4HH, UK; b York Neuroimaging Centre, University of York, York Science Park, York, YO10 5DG, UK; c Department of Psychology, University of York, York, YO10 5 DD, UK

**Keywords:** Mate choice, Sexual selection, Physical attractiveness, Body mass index, Ebbinghaus illusion, Size-contrast illusion

## Abstract

Could signallers use size contrast illusions to dishonestly exaggerate their attractiveness to potential mates? Using composite photographs of women from three body mass index (BMI) categories designed to simulate small groups, we show that target women of medium size are judged as thinner when surrounded by larger women than when surrounded by thinner women. However, attractiveness judgements of the same target women were unaffected by this illusory change in BMI, despite small true differences in the BMIs of the target women themselves producing strong effects on attractiveness. Thus, in the context of mate choice decisions, the honesty of female body size as a signal of mate quality appears to have been maintained by the evolution of assessment strategies that are immune to size contrast illusions. Our results suggest that receiver psychology is more flexible than previously assumed, and that illusions are unlikely to drive the evolution of exploitative neighbour choice in human sexual displays.

## 1 Introduction

Evolutionarily stable sexual signals must provide honest information about a signaller’s underlying qualities to some receivers, at least some of the time (Johnstone, 1998; Searcy & Nowicki, 2005; Rowell, Ellner, & Reeve, 2006). The honesty of a signal can be ensured either if it has high costs that prevent low quality cheaters from exploiting the fitness benefits associated with communicating high quality, or if it is physically constrained to be impossible to fake (Zahavi, 1975; Grafen, 1990; Maynard Smith & Harper, 2003). Physical size is a common attribute of sexual signals, presumably either because only high quality individuals can pay the increased costs associated with developing and maintaining larger signals, or because size is particularly hard to fake. Indeed, body size has been held up as an example of a signal where deception is impossible (Rowell et al., 2006). Although individuals can exaggerate their body size using ploys such as raising their hair or adopting flattering colour patterns, the deceptive advantage derived from such exaggerators will be evolutionarily short-lived because they will rapidly spread to fixation (Maynard Smith & Harper, 2003). However, the situation will not be so straightforward when exaggeration arises from illusions generated by comparison with other individuals, because signallers can potentially choose their immediate neighbours strategically (Bateson & Healy, 2005; Callander, Jennions, & Backwell, 2011). Indeed, Gasparini, Serena, and Pilastro (2013) argue that male guppies (*Poecilia reticulate*) choose females already surrounded by drab, less attractive males rather than females surrounded by bright, more attractive males because being relatively more attractive than the competition is likely to increase their mating success.

The visual illusion of size contrast is exemplified by the well-known Ebbinghaus illusion (also known as Titchener circles) in which the apparent size of a central target circle is altered by the size of surrounding inducer circles (Ebbinghaus, 1902). This illusion also occurs when the targets and inducers are of dissimilar shapes (Coren & Miller, 1974; Rose & Bressan, 2002) and when they are complex stimuli such as images of human faces (Stapel & Koomen, 1997), suggesting that size contrast could also be relevant to the perception of any size-based sexual signal. This phenomenon therefore raises problems for the honesty of size-based signals, because a signaller could use it to enhance their attractiveness at minimal cost simply by positioning themselves in a flattering group (Bateson & Healy, 2005). So why has physical size been maintained as a common signal of mate quality by sexual selection if it can so easily be exaggerated?

Here we ask whether size contrast illusions occur in the perception of a natural sexual signal for which the primary determinant of attractiveness is known to be size, namely the human female body. In human females of reproductive age, physical size, as measured by body mass index (BMI), is the primary predictor of physical attractiveness judgements (Tovée, Maisey, Emery, & Cornelissen, 1999; Rilling, Kaufman, Smith, Patel, & Worthman, 2009; Tovée, Edmonds & Vuong, 2012). For example, Tovée et al. (1999) found that BMI accounted for over 70% of the variance in a regression model of their participants’ attractiveness judgements. A result found to be cross-culturally consistent (e.g. Swami & Tovée 2005; Tovée, Swami, Furnham, & Mangalparsad, 2006). In Western populations, women are optimally attractive with a BMI of 19–20, and attractiveness declines as BMI increases (Tovée et al., 1999). If size contrast occurs in the judgement of female BMI, then a woman could reduce her perceived BMI, and therefore increase her judged attractiveness, merely by positioning herself in close proximity to higher BMI, less attractive women. To date, studies investigating the relationship between BMI and attractiveness have employed judgements of isolated images of female bodies, precluding the possibility of size contrast effects. However, since there is a strong positive relationship between BMI and the dimensions of the human female body in a two dimensional photograph (e.g. correlations between BMI and Perimeter–Area Ratio (PAR); Tovée et al., 1999), the potential exists to explore size contrast effects in attractiveness judgements using the standard methodology of body attractiveness studies.

**Figure 1:**
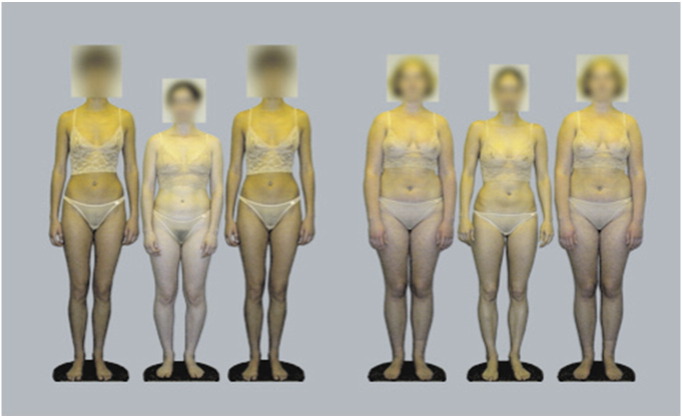
A typical image array as seen by a subject in a single trial of the experiment. This image has the form LM_L_L_HM_R_H because the low BMI inducers are in the left-hand group.

For the current experiments we designed stimuli consisting of composites of photographs of real women. Each composite picture featured two groups of three women standing side by side. The body sizes of the six women were chosen to replicate the contrasts present in stimuli used to illustrate the standard Ebbinghaus illusion. The six women came from three BMI classes designated low, medium and high. The central women in the two groups were non-identical women of medium BMI (the ‘targets’: M_L_ and M_R_). These target women were flanked in one case by two women of lower BMI, and in the other by two women of higher BMI (the ‘inducers’: L and H), resulting in an image of the form LM_L_L_HM_R_H or HM_L_H_LM_R_L (see Fig. 1). Observers viewed a series of different stimuli of this form and were instructed to make a judgement of which of the two targets present in each image they perceived as either larger (size judgement condition) or more attractive (attractiveness judgement condition).

In Experiment 1 male and female observers each completed both judgement conditions. If a typical size contrast effect is observed, then we predicted that the target woman flanked by the higher BMI inducers should be judged as both thinner and more attractive than the target woman flanked by the lower BMI inducers. Experiment 2 was a between-subjects replicate of Experiment 1 in which we additionally tracked the eye movements of observers in order to understand how they viewed the composite images when making their judgements.

## 2 Materials and methods

### 2.1 Stimuli

The digital photographs of young women (mean age 20.7 years, s.d. 2.2 years) used for the current experiment were a subset of 18 from a larger set collected by Smith et al. (Smith, Cornelissen, & Tovée, 2007) where details of stimulus collection and skin tone quantification are described. The volunteers’ heads were blurred in the resulting images to ensure anonymity and also remove any confounding effects of facial attractiveness on subsequent judgements of attractiveness. The photographs were chosen such that there were six images from each of three BMI classes: low (18.4–19.2), medium (22.0–22.7) and high (25.3–26.7). We have previously shown that Western observers reliably rate women from the low class as more attractive than women from the medium class, and women from the medium class as more attractive than women from the high class (Tovée, Reinhardt, Emery, & Cornelissen, 1998; Tovée et al., 1999). For the set of 18 bodies used in this study, the area covered by each body in the photograph was measured using Imagej ( http://imagej.nih.gov/ij/). In this set, BMI is correlated with the area each body covers in the digital photograph (Pearson correlation; r = 0.70, p < 0.001) and height is not correlated with body area (Pearson correlation; r = − 0.14, p = 0.592). This shows that the size of the body is proportional to its BMI, consistent with our use of BMI to guide our image selection in our version of the Ebbinghaus illusion.

For the purposes of this experiment, the images obtained above were arranged into groups of three. The central target image of each group was always an image of a woman with a medium BMI. The two flanking images in each group were identical inducers from either the low or high BMI class. To verify that our stimuli were consistent with standard presentations of the Ebbinghaus illusion, we checked that the area covered by the bodies was significantly different between the three BMI classes (one-way ANOVA, F_2,15_ = 8.2, p < 0.005). In each trial the observer was simultaneously presented with two such groups of three images. In one group the inducers were always of lower BMI and in the other the inducers were always of higher BMI (see Fig. 1 for an example). The entire array of six images was presented on a 21” LCD display (1600 × 1200 native resolution; 32-bit colour depth), and subtended ~ 8º horizontally and ~ 4º vertically at a viewing distance of 2 m.

In each trial the two target images were one of the 15 possible pairs of the six medium BMI images (a target image was never paired with itself). Each of these pairs of targets was paired with a randomly chosen pair of inducer stimuli, one from the high BMI group and one from the low BMI group. The arrangement of the images on the screen was counterbalanced such that each target and each inducer appeared on both the left and right sides of the screen giving a total of four possible arrangements for each target–inducer pair. This design resulted in a total of 60 possible trials (15 target pairs × 4 possible permutations).

### 2.2 Subjects and procedure

Experiments 1 and 2 were approved by the ethics committee of the Psychology Department, at York University. In Experiment 1 60 University of York undergraduates (30 males, 30 females) served as subjects (mean age: 20.46 years, s.d. 0.68). The subjects made a series of two-alternative forced choices (2AFCs) between the two central target images in each group of three images. We used a within-subjects design: each subject made judgements about both attractiveness and body size. In the attractiveness judgement condition subjects were asked to choose which of the two target females appeared the most attractive, whereas in the size judgement condition they were asked to decide which of the two target females appeared to be of the larger body size. We specifically explained to each participant that by size we meant body fat, and that we wanted them to choose the body that appeared fattest to them. Each trial comprised the same sequence of events: a uniform grey screen (1000 ms) was followed by a central fixation cross (1000 ms) and then the image array (3500 ms). Once the images had disappeared, subjects were asked to report whether they thought the left or right target was more attractive/larger. There was no time limit for the response. Following a response the next trial was initiated. Each subject completed all 60 possible trials in one judgement condition (size or attractiveness) followed by a further 60 trials in the other condition. The order of trials was randomly chosen and different for each subject, and the order in which subjects did the attractiveness and body size judgement conditions was counterbalanced. Subjects were given 3–4 practice trials, for which data were not recorded, prior to starting the 60 experimental trials (for each condition), in order to ensure that they understood the task.

In Experiment 2, 96 University of York undergraduates (48 males, 48 females) served as subjects (mean age: 20.54, s.d. 2.2 years). Half of the subjects of each sex were randomly assigned to the attractiveness judgement condition and the remainder did the size judgement condition. The between-subjects design minimized image familiarity ensuring that no recognition components could bias subjects’ eye-movements towards a different pattern.

The 2AFC procedure was similar to that employed in Experiment 1 but with the addition of eye tracking. Subjects sat at a desk in a darkened room (viewing distance of 60 cm). Their head was restrained by a forehead and chin rest, and the pupil tracking camera focused. Subjects completed a total of 60 trials divided into 3 blocks of 20. Each block consisted of the following sequence of events. At the start of each block the eye-tracker was calibrated. Then, for each trial, a central fixation dot appeared (2000 ms), followed by the image array (3500 ms). This was replaced by a uniform grey screen until the observer made his or her judgement by button press. There was no time limit for the response. Following a response the next trial was initiated with the central fixation dot reappearing. Each block lasted approximately 450 s.

### 2.3 2AFC analysis

We predicted that targets flanked by high BMI inducers should have a lower probability of being chosen as larger than those flanked by low BMI inducers. Similarly, targets flanked by high BMI inducers should also have a higher probability of being rated as more attractive than those flanked by low BMI inducers. As a conservative preliminary test of these effects we computed the proportion of trials on which each observer rated the target flanked by the low BMI inducers as the largest or the high BMI inducers as the most attractive (depending on condition), and used a one-sample t-test to compare the resulting 60 proportions against a null expectation of 0.5.

To explore the data further, and control for expected confounds we used generalised linear modelling. We arbitrarily assigned the probability that an observer responded that the right hand target in the array had the larger body size (for body size judgements) or was the most attractive (for attractiveness judgements) as our dependent variable, and modelled how this probability was affected by various features of the target and inducer images. To capture the effect of inducer BMI we computed the difference in BMI between the right and left hand inducers (right inducer BMI − left inducer BMI = ∆BMI_i_); a positive value of ∆BMI_i_ means that the right inducers were larger than the left inducers. According to the size contrast hypothesis, positive values of ∆BMI_i_ should make the right target appear thinner thereby increasing the probability of the right target being rated as more attractive. Negative values of ∆BMI_i_ mean that the left inducers were larger than the right inducers, and this should increase the perceived size of the target on the right, thereby reducing the probability of the right target being rated more attractive.

In addition, we controlled for potentially confounding effects due to differences between the targets themselves. We know from ratings of isolated images that BMI and skin tone influence judgements of attractiveness (Smith et al., 2007). Since the two targets were images of different individuals, it is plausible that small differences between the BMI and skin-tone of the targets could influence attractiveness judgements. Differences in target BMI and skin tone were calculated as left target BMI − right target BMI (∆BMI_t_) and right target skin tone − left target skin tone (∆ST_t_) respectively. In both cases, positive values of ∆BMI_t_ and ∆ST_t_ would be associated with an increased probability of rating the right hand target as the more attractive.

To test for main effects of inducer BMI (∆BMI_i_) and observer sex, while controlling for any confounding effects of target BMI (∆BMI_t_) and target skin tone (∆ST_t_), and accounting for repeated measures within observers, we used PROC GLIMMIX v9.2 in SAS (SAS Institute, North Carolina, USA) to fit a generalized linear mixed model. We assumed a binomial distribution for the response variable and used the logistic link function. For completeness, we fitted a full model with all possible interaction terms.

### 2.4 Eye-tracking data capture and analysis

We used Cambridge Research Systems (CRS) Video eye-tracker toolbox for MATLAB v7.7 programme using ViSaGe Software Library v8.1. This was interfaced with a CRS CT6 response box and ViSaGe response tracker which controlled timing of the experimental sequence, registered observers' behavioural responses and controlled the CRS VET 50Hz eye-tracker. The eye-tracker tracked the horizontal and vertical locations of the observer's left pupil whilst the stimuli were present on a CRT Mitsubishi Diamond Pro 2070 monitor (88 Hz vertical refresh rate).

Eye movements were recorded for the duration of each trial, but we did not include the first 300 ms in our analysis, as otherwise this would include the initial fixation which is determined by the observer having to fixate the cross. We constructed a sampling grid of square cells (0.5° × 0.5° each), and applied it across the entire image (see Cornelissen, 2009). We modeled differences in fixation counts between conditions using GLMMs.

The most important outcome from the statistical modelling was to identify where in the images of women the observers looked more or less frequently comparing attractiveness to body size. To do this, we computed the predicted population margins from the GLMMs and compared them using tests for simple effects by partitioning the interaction effects, controlling for multiple comparisons. Ultimately, therefore, we compared the fixation count in corresponding cells of the sampling grid between the two kinds of judgement, separately for male and female observers, controlling for spatial correlation and multiple comparisons.

## 3 Results

All of the raw data analysed in this section are available as online supplementary information accompanying this paper.

In Experiment 1, if a typical size contrast illusion occurred, then we predicted that the target woman flanked by the higher BMI inducers should be judged as both thinner and more attractive than the target woman flanked by the lower BMI inducers. As predicted, there was an overall significant effect of the BMI of the inducer women on the judged body size of the target women: observers rated a medium BMI target as smaller more often when she was flanked by high BMI inducers than when she was flanked by low BMI inducers (one-sample t-test, t = 2.44(59), p < 0.05). Counter to our predictions however, there was no significant effect of the BMI of the inducers on the judged attractiveness of the target images (one-sample t-test, t = 1.06(59), p > 0.1). These main effects were replicated in a single general linear model in which we modelled the effects of ∆BMI_i_, ∆BMI_t_, ∆ST_t_, observer sex and judgement type (size or attractiveness). For Experiment 1 we found significant main effects of ∆BMI_i_ (F_1,7191_ = 12.93, p < 0.01), ∆BMI_t_ (F_1,7191_ = 144.88, p < 0.01) and ∆ST_t_ (F_1,7191_ = 253.09, p < 0.01), but no significant main effect of sex or judgement type (F_1,7191_ = 0.08, p = 0.78 and F_1,7191_ = 0.81, p = 0.37). Critically, there was a significant interaction between ∆BMI_i_ and judgement type (F_1,7191_ = 23.68, p < 0.01), showing that there are differences in the effect of ∆BMI_i_ on the two judgements. To characterize in detail the nature of the interaction between judgement type and ∆BMI_i_ we ran separate analyses for the body size and attractiveness judgements.

**Figure 2:**
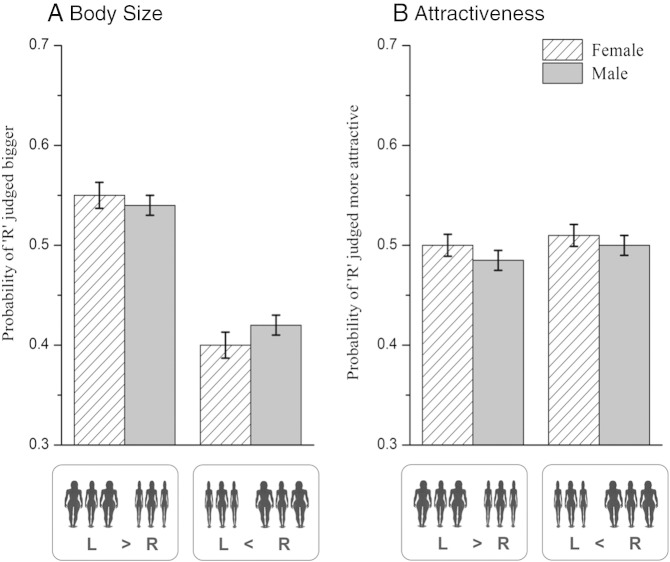
(A) Bar chart showing the probability of the right-hand target being judged as having a larger body size than the left-hand target in two situations: first when the left inducers were larger than the right inducers (left of x-axis); second when the left inducers were smaller than the right inducers (right of x-axis). (B) Bar chart showing the probability of the right-hand target being judged more attractive than the left-hand target in the same two situations. The data are displayed separately for female (cross hatched) and male (solid grey) observers. The probability values have been corrected for ∆BMI_i,_ ∆BMI_t_ and ∆ST_t_. Error bars represent ± 1 standard error.

Fig. 2A shows the average probability of choosing the right hand target as the larger body size corrected for ∆BMI_t_ and ∆ST_t_. Running the general linear model with the size judgement as the outcome variable, we found significant main effects of ∆BMI_i_ and ∆BMI_t_ (F_1,3592_ = 46.36, p < 0.01and F_1,3592_ = 767.69, p < 0.01 respectively), but no significant main effect of sex or ∆STt (F_1,3592_ = 0.01, p = 0.91 and F_1,3592_ = 3.02, p = 0.08 respectively). Fig. 2B shows the average probability of choosing the right hand target as the more attractive corrected for ∆BMI_t_ and ∆ST_t_. Running the general linear model with the attractiveness judgement as the outcome variable, we found significant main effects of ∆BMI_t_ and ∆ST_t_ (F_1,3592_ = 216.22, p < 0.01 and F_1,3592_ = 424.62, p < 0.01 respectively) on attractiveness, but no significant effect of sex or ∆BMI_i_ (F_1,3592_ = 0.84, p = 0.36 and F_1,3592_ = 0.32, p = 0.57 respectively).

In Experiment 2, analysis of the judgement data replicated the main results reported above. We again used a general linear model in which we included the effects of ∆BMI_i_, ∆BMI_t_, ∆ST_t_, sex and judgement type. We found significant main effects of judgement type (F_1,5553_ = 19.67, p < 0.01) and ∆BMI_t_ (F_1, 5553_ = 184.93, p < 0.01) and ∆ST_t_ (F_1, 5553_ = 253.09, p < 0.01 respectively), but no significant main effect of sex or ∆BMI_i_ (F_1, 5553_ < 0.01, p = 0.93; F_1, 5553_ = 0.61, p = 0.44 respectively). Critically, there was again a significant interaction between ∆BMI_i_ and judgement type (F_1,5553_ =4.02, p = 0.04), showing that there are differences in the effect of ∆BMI_i_ on the two judgements. To characterize in detail the nature of the interaction between task and ∆BMI_i_ we again ran separate analyses for the body size and attractiveness judgements.

For body size judgements, we found significant main effects of ∆BMI_i_, ∆BMI_t_ and ∆ST_t_ (F_1,2712_ = 6.47, p = 0.01; F_1,2712_ = 759.25, p < 0.01 and F_1,2712_ = 126.41, p < 0.01 respectively), but no effect of sex (F_1,2712_ = 0.25, p = 0.61). For attractiveness judgements, we found significant main effects of ∆BMI_t_ and ∆ST_t_ (F_1,2832_ = 154.58, p < 0.01 and F_1,2832_ = 470.07, p < 0.01 respectively), but again no significant effect of sex or ∆BMI_i_ (F_1, 2832_ = 0.41, p = 0.52 and F_1, 2832_ = 1.09, p = 0.30 respectively).

**Figure 3:**
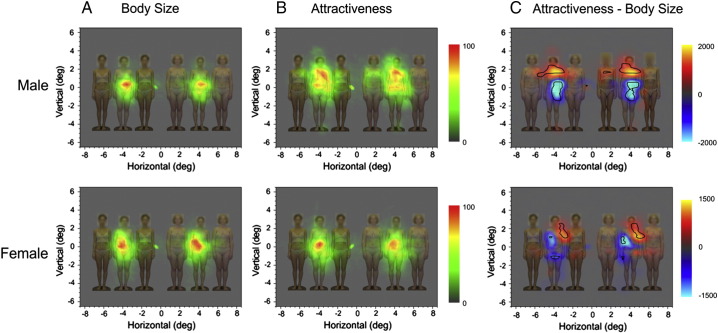
Eye-tracking results. (A and B) Contour plots of the fixation distributions for the attractiveness and body size judgement conditions for both sexes overlaid onto a typical stimulus array. In order to facilitate inspection of the data across all conditions, fixation density in the left and central columns has been converted to a percentage score, indicated by colour bars, with red indicating the highest density. (C) The differences in the fixation density (i.e. differences in raw scores) between attractiveness and body size judgement. Positive differences are shown as red/yellow colours; negative differences are shown as blue/cyan colours. The black contours demarcate regions within which these differences are statistically significant (p < 0.05).

The results of the eye-tracking are summarised in the maps of fixation distributions shown in Fig. 3. We found no evidence for any difference in distribution of fixations to the inducers between the two judgement conditions. However, there was a difference in looking patterns between the two conditions. Observers tended to focus on the waist in the size judgement condition (Fig. 3A), but additionally included other areas of the upper torso in the attractiveness condition (Fig. 3B). As a result, observers spent significantly more time looking at the chest region and less time looking at the waist region in the attractiveness judgement condition compared to the size judgement condition (Fig. 3C).

## 4 Discussion

Using composite digital photos simulating groups of three women we have shown that average-sized women are judged as thinner when surrounded by larger women than when surrounded by thinner women. However, attractiveness judgements of the same women were unaffected by their context, despite body size being the primary determinant of attractiveness of female bodies. In support of previous work (Tovée et al., 1999; Smith et al., 2007), we also found that the BMI and the skin tone of the target women themselves contributed to their judged size and attractiveness with thinner, more tanned women being judged as smaller and more attractive. Thus, in a situation in which a size contrast illusion occurs, we have demonstrated that judgements of female physical attractiveness are influenced only by the attributes of a woman herself, and not by the social context in which she is viewed.

To increase the ecological validity of our stimuli we did not scale the heights of the women to be identical. It is therefore possible that the height of the women might have played a role in the size judgements. However, we specifically asked our participants which image appeared fatter, not taller. This cued the participants to specifically attend to this aspect of the bodies, which is confirmed by the pattern of eye-movements (see Fig. 3). The participants’ fixations are centred on the middle of the body and show eye-movement across the stomach, consistent with judging body fat rather than height (which would require eye-movements along the length of the bodies). Another potential flaw might be that the lower BMI bodies tended to be taller. If the taller images are also the low BMI images, then analysing the results for the effect of inducer BMI, one might see an effect based on height rather than BMI. However, if height were responsible for the results we would see the low BMI flanked targets as “smaller” and the high BMI flanked target as “larger”. We find the opposite pattern of results. The low BMI flanked target bodies are seen as larger and the high BMI flanked images as smaller. Therefore, we are confident that the results from the size judgement task are driven by the relative BMIs of the bodies and not their heights.

Was the change in the perceived size of the target women in our size judgement condition potentially sufficient to produce a change in perceived attractiveness? Previous data on the Ebbinghaus illusion show that across a range of similarly and dissimilarly shaped targets and inducers, the size induction perceived is in the region of 6% (Coren & Enns, 1993; Rose & Bressan, 2002). This figure is substantially larger than the difference in BMI between the target women necessary to produce a difference in attractiveness judgements. In our experiment, the maximum difference in BMI between the target women was only 2.97% (occurring when the highest and lowest BMI women in the medium class were paired), yet our analysis indicated that the difference in BMI between the two target women explained a significant proportion of the variance in attractiveness judgements in both our experiments. Thus, the change in apparent body size produced by the size-contrast illusion should be of sufficient magnitude to trigger an attractiveness preference between the target women. Given this conclusion, our findings raise the question of why it is that a size contrast illusion occurs in the domain of body size judgements but not in the domain of body attractiveness judgements. We discuss the proximate and ultimate explanations for this dissociation.

Our eye-tracking results provide behavioural evidence supporting the hypothesis that observers engaged different assessment strategies when making size and attractiveness judgements. Specifically, observers tended to focus on the waist in the size judgement condition, but additionally looked at other areas of the torso, and specifically the chest area, in the attractiveness condition. A similar difference has previously been reported in eye-tracking during size and attractiveness judgements of single female bodies suggesting that it is a robust finding (Cornelissen, Hancock, Kiviniemi, George, & Tovée, 2009). This difference suggests that when asked to judge attractiveness, observers are seeking different information than when asked to judge size, but it is not clear why these different looking patterns should lead to differences in the magnitude of the size-contrast illusion perceived. Our motivation for the eye tracking was the hypothesis that observers might look less at the inducer women when judging attractiveness than when judging size and hence perceive a reduced illusion. However, the eye-tracking data revealed no difference in fixations to the inducer women between the two judgement conditions. In order to explain the lack of the illusion in the attractiveness judgement condition we turn instead to what is known about the psychological and neural mechanisms underlying the Ebbinghaus illusion.

Previous experiments have demonstrated that the Ebbinghaus illusion is not an inescapable constraint of perceptual systems. The magnitude of the illusion produced by a given stimulus array is not a fixed feature of that stimulus array, but is instead amenable to external sources of information available to the observer. For example, an identical array of images of faces produced a greater illusion when observers were told that the faces represented fraternity or sorority members compared with when they were told that they represented men or women born in the month of May (Pickett, 2001). The explanation given for this effect is that the magnitude of the Ebbinghaus illusion is determined by the degree to which observers view the stimulus set as an integrated whole as opposed to a collection of unconnected individual components (Coren & Enns, 1993; Pickett, 2001); in the above example, the stimulus is seen as a group of friends as opposed to a set of individuals with an arbitrary connection. Further support for this hypothesis comes from the fact that autistic individuals, who are argued to be low in central coherence – the tendency to process incoming information in its context – are less susceptible to the Ebbinghaus illusion than normal controls (Happe, 1996). Therefore, it is possible that in asking our observers to judge attractiveness rather than size we caused them to consider the women in the pictures as individuals rather than as groups, and in so doing eliminated the illusion.

Attractiveness judgements are a prerequisite for mate choice decisions, and therefore imply action as opposed to mere perception. Much evidence supports a ‘two stream’ hypothesis whereby the visual processing involved in preparing for action is conducted by a different neural pathway from that involved in simple identification of objects. The dorsal “action” stream transforms incoming visual information into coordinates for motor behaviour, whereas the ventral “perceptual” stream is involved in identification of objects. Intriguingly, there is evidence that whilst the ventral stream uses relative metrics and scene based frames of reference, the dorsal stream uses absolute metrics and ego-centric frames of reference (Goodale, 2011; Kravitz, Saleem, Baker, & Mishkin, 2011). For example, when observers are asked to judge the size of the central circles in an Ebbinghaus illusion they perceive the illusion, but when asked to grasp the circles their grip size is accurate (Aglioti, Desouza, & Goodale, 1995). It is therefore possible that in asking observers to judge attractiveness we are engaging the mechanisms underlying vision-for-action as opposed to merely vision-for-perception. Thus, there is evidence from the psychology literature that the Ebbinghaus illusion is suppressed when observers consider the targets as unconnected individuals and when they are planning an action. It seems plausible that if attractiveness judgements have evolved to guide mate-choice decisions, then when making these judgements, observers should consider the target women as individual prospective mates, and in so-doing, engage mechanisms that deliver accurate information on body size.

Although we have couched our discussion so far in terms of mate choice, we tested both male and female participants in our experiments and found no difference in their judgements. Why should women judging women show similar patterns to those seen in men judging women? Mate choice theory predicts that an individual will have a precise and accurate idea of what the opposite sex find attractive (Buss, 2003). This allows them to judge their own relative value, with respect to their peer group, and match this value with the value of a prospective mate. Thus, mate choice theory predicts that there will not be any difference between men and women in their ideal size for the female body. There is evidence to support this hypothesis in attractiveness studies which have suggested the same ideal female body size is held by both sexes (e.g. Tovée, Hancock, Mahmoodi, Singleton, & Cornelissen, 2002, Tovée et al., 1999, 2006; Crossley, Cornelissen, & Tovée, 2012). We would argue therefore, that both sexes are potentially targets for this illusion in the context of mate choice, males, as it effects their choice of partner, and females as it effects their perception of their competitors and their relative standing with respect to them.

From an evolutionary perspective it is easy to understand why attractiveness judgements, and hence mate-choice decisions should be immune to illusions. To be evolutionarily stable, a sexual signal must on average provide honest information and dishonest signals will be eliminated by natural selection (Johnstone, 1998; Searcy & Nowicki, 2005; Rowell et al., 2006). In the search for the evolutionary mechanisms responsible for signal honesty, evolutionary biologists have focussed on features of signals that ensure honesty, namely their costs. However, our current results suggest that the assessment strategies evolved by signal receivers could play an equally important role in the evolutionary arms race. We predict that natural selection should favour strategies for assessing sexual signals that are immune to illusions generated by the context in which a signal is received. Further work is needed to understand why it is that in some species individuals are apparently able to exploit contrast effects to enhance their attractiveness in mate choice contexts (e.g. Callander et al., 2011; Gasparini et al., 2013).

Behavioural ecologists have long argued for the importance of receiver psychology in driving the evolution of biological signals (Guilford & Dawkins, 1991; Ryan, Fox, Wilczynski, & Rand, 1992). The idea that animals could exploit perceptual illusions in signalling is regularly mooted (Guilford & Dawkins, 1993; Zahavi & Zahavi, 1997; Bateson & Healy, 2005; Kelley & Endler, 2012), and laboratory experiments showing that species other than humans can be fooled by optical illusions (Fujita, Blough, & Blough, 1991; Pepperberg, Vicinay, & Cavanagh, 2008) have been cited as evidence supporting this possibility(Kelley & Endler, 2012). However, in demonstrating that whether or not an illusion is seen depends on the type of judgement being made, we provide evidence that the psychology of signal receivers is unlikely to be a fixed constraint driving signal evolution. If signal receivers can adopt signal assessment strategies that suppress illusions, and hence gain more accurate information about the quality of signallers, then natural selection should have favoured these strategies in the context of signal assessment. In the case of size contrast illusions, we have presented evidence that receiver psychology is unlikely to be a fixed constraint of human signal receivers in a mate choice context.

## Supplementary Materials

The following are the Supplementary data to this article.

behavioural_data_README.txt

behavioural_data.txt

eye_movement_README.txt

eye_movement_data.txt

## Supplementary Materials

Supplementary data to this article can be found online at http://dx.doi.org/10.1016/j.evolhumbehav.2013.11.007.
